# Editorial—Special Issue “Nutraceuticals in Human Health and Disease”

**DOI:** 10.3390/ijms19041213

**Published:** 2018-04-17

**Authors:** Leticia M. Estevinho

**Affiliations:** CIMO-Mountain Research Centre, Agricultural College of Bragança, Polytechnic Institute of Bragança, Campus Santa Apolónia, E-5301-855 Bragança, Portugal; leticia@ipb.pt; Tel.: +351-273-303-342; Fax: +351-273-325-405

The term “nutraceutical”, derived from the words “nutrition” and “pharmaceutical” was coined in 1989 to describe substances that could be used as foods that possess health benefits. The range of nutraceutical products is widely diverse and may be divided into three main categories: (i) natural products, comprising, among others, herbs and spices; (ii) dietary supplements; and (iii) functional foods. Based on food sources, nutraceuticals can be classified as: (i) dietary fibres of plant origin; (ii) probiotics, which are live microbial feed supplements; (iii) prebiotics, dietary ingredients that selectively alter the composition or metabolism of gut microbiota; (iv) polyunsaturated fatty acids, omega-3 or omega-6 fatty-acids; (v) antioxidant vitamins, including vitamin C, vitamin E and carotenoids; (vi) biologically active phytochemicals like polyphenols; and (vii) spices, whose main components are terpenes and other constituents of essential oils.

In the recent years, the field of nutraceuticals has progressed from being a mere conceptual area within biomedical research to a value-added industry with a promising future ahead. Indeed, lately, both the academic community and the general population have become increasingly interested in the health benefits and potential risks pertaining to beverages and food products beyond their basic nutritional assets. In addition, continuing scientific research has provided evidence regarding the biologically active compounds and underlying physiological mechanisms, highlighting the importance of nutraceuticals as complements to the conventional therapies and medications allowing dose reduction and decreasing the occurrence of adverse effects. Despite the undeniable progress in the field of nutraceuticals, there are still several issues that remain to be addressed. These include clinical evidence supporting in vitro claims, regulatory aspects and assurance of nutraceuticals’ identity, quality and safety.

The special issue “Nutraceuticals in Human Health and Disease” in the *International Journal of Molecular Sciences* comprises one review article and 16 original research papers that provide important insights into the state of the nutraceuticals field. Nosrati et al. [1] review the molecular mechanisms and pathways underlying the cancer prevention potential of dietary compounds and present information regarding the roles of microRNAs and epigenetic modifications in cancer initiation. Chobot et al. [2] use mixed electrochemical and redox chemical method to assess pro and antioxidant activities of three flavonoids. Seto et al. [3] characterize the ability of a standardised three-herb formulation to inhibit hydrogen peroxide-induced oxidative damage in cultured human vascular endothelial cells and the associated mechanism appears to be related to the inhibition of the apoptotic cascade. Pinteus et al. [4] investigate the antioxidant and cytoprotective activities of antioxidant-enriched fractions from the common seaweed *Fucus spiralis* in a human cell in vitro model (MCF-7 cells). Han et al. [5] report the atheroprotective effects of an ethanol extract of soy leaf via modulation of Krüppel-like factor 2 and adhesion molecules, suggesting that supplementation with such extract is promising for preventing HCD-induced atherosclerosis effectively.

The studies conducted by Forney et al. [6] provide evidence that dietary quercetin attenuates adipose tissue expansion and inflammation and alters adipocyte morphology in a tissue-specific manner. Giuseppe et al. [7] describe a randomized placebo-controlled trial to assess the effects of mesoglycan supplementation on endothelial damage and walking distance in diabetic patients with peripheral arterial disease; preliminary data are interesting and open doors for further investigation. Spigoni et al. [8] perform a randomized placebo-controlled trial assessing the effects of a nutraceutical formulation comprising berberine, red yeast rice and chitosan on the non-HDL cholesterol levels. Trombetta et al. [9] evaluate the polyphenols and α-tocopherol content and the antioxidant capacity of some early and late harvest organic and non-organic mono- and multi-varietal extra virgin olive oils. Araújo et al. [10] evaluate the phenolic compounds and fatty acid composition of bee pollen extracts and assessed their antioxidant activity and enzyme-inhibitory potential. Santos et al. [11] study the chemical profile and several biological activities of stingless bees’ geopropolis. Silihe et al. [12] report the antitumor potential of *Ficus umbellata* constituents that appears to be due to apoptosis induction through the ROS-dependent mitochondrial pathway. The work conducted by Dai et al. [13] reveals that oxymatrine, an alkaloid compounds extracted from the root of *Sophora flavescens*, inhibits influenza A virus replication and possesses anti-inflammatory activities and that the underlying mechanism may be linked to its ability to inhibit IAV-induced activations of the TLR4, p38 MAPK, and NF-κB pathways.

Studies developed by Liu and Dey [14] suggest that dietary phenethyl isothiocyanate may slow down colon carcinogenesis, what is potentially associated with epigenetic modulation of NFκB1 signalling. Trejo et al. [15] highlight that the beneficial effects of allicin in chronic kidney disease, reducing hypertension and oxidative stress and improving renal dysfunction, comparable to those of losartan. Zhou et al. [16] evaluate the effects of *Acanthopanax senticosus* in repressing radiation-induced protein expression changes on prefrontal cortex, and suggest that this compound may be a promising alternative medicine for the treatment of radiation injury in the brain yet further testing are needed to understand whether the bioactive ingredients could be effective through the blood–brain barrier. Klimaszewska-Wiśniewska et al. [17] provide preliminary evidence regarding the synergistic anticancer efficacy of the combination of fisetin with paclitaxel on non-small cell lung cancer cells.

Collectively, these studies support a role for nutraceuticals in the prevention and amelioration of several diseases. Their innovation in the field and their successful future clinical application will be governed by evidence regarding safety, purity and efficacy. The use of these compounds in clinical practice is emerging, yet important pharmaceutical and clinical issues remain to be answered.
